# Gene Expression Profiles Reveal Extracellular Matrix and Inflammatory Signaling in Radiation-Induced Premature Differentiation of Human Fibroblast *in vitro*

**DOI:** 10.3389/fcell.2021.539893

**Published:** 2021-02-18

**Authors:** Carsten Herskind, Carsten Sticht, Ahmad Sami, Frank A. Giordano, Frederik Wenz

**Affiliations:** ^1^Cellular and Molecular Radiation Oncology Laboratory, Department of Radiation Oncology, Universitaetsmedizin Mannheim, Medical Faculty Mannheim, Heidelberg University, Mannheim, Germany; ^2^Centre for Medical Research, Medical Faculty Mannheim, Heidelberg University, Mannheim, Germany; ^3^Department of Radiation Oncology, Universitaetsmedizin Mannheim, Medical Faculty Mannheim, Heidelberg University, Mannheim, Germany

**Keywords:** fibroblast differentiation, extracellular matrix, radiation-induced fibrosis of the skin, inflammatory signaling, cell cycle-related genes

## Abstract

**Purpose:**

Fibroblasts are considered to play a major role in the development of fibrotic reaction after radiotherapy and premature radiation-induced differentiation has been proposed as a cellular basis. The purpose was to relate gene expression profiles to radiation-induced phenotypic changes of human skin fibroblasts relevant for radiogenic fibrosis.

**Materials and Methods:**

Exponentially growing or confluent human skin fibroblast strains were irradiated *in vitro* with 1–3 fractions of 4 Gy X-rays. The differentiated phenotype was detected by cytomorphological scoring and immunofluorescence microscopy. Microarray analysis was performed on Human Genome U133 plus2.0 microarrays (Affymetrix) with JMP Genomics software, and pathway analysis with Reactome R-package. The expression levels and kinetics of selected genes were validated with quantitative real-time PCR (qPCR) and Western blotting.

**Results:**

Irradiation of exponentially growing fibroblast with 1 × 4 Gy resulted in phenotypic differentiation over a 5-day period. This was accompanied by downregulation of cell cycle-related genes and upregulation of collagen and other extracellular matrix (ECM)-related genes. Pathway analysis confirmed inactivation of proliferation and upregulation of ECM- and glycosaminoglycan (GAG)-related pathways. Furthermore, pathways related to inflammatory reactions were upregulated, and potential induction and signaling mechanisms were identified. Fractionated irradiation (3 × 4 Gy) of confluent cultures according to a previously published protocol for predicting the risk of fibrosis after radiotherapy showed similar downregulation but differences in upregulated genes and pathways.

**Conclusion:**

Gene expression profiles after irradiation of exponentially growing cells were related to radiation-induced differentiation and inflammatory reactions, and potential signaling mechanisms. Upregulated pathways by different irradiation protocols may reflect different aspects of the fibrogenic process thus providing a model system for further hypothesis-based studies of radiation-induced fibrogenesis.

## Introduction

Fibroblasts are the most abundant cells in connective tissue and are the major source of extracellular matrix (ECM) proteins. Thus fibroblasts are considered to play a major role in the development of fibrotic reaction after radiotherapy. Fibroblasts show a limited proliferation capacity *in vitro* (the socalled ‘Hayflick limit’) which was initially thought to correlate with the age of donors (Hayflick, 1965, 1980). However, no correlation was found in a large longitudinal study when only healthy patients were included in the analysis (Cristofalo et al., 1998). In fact, heterogeneity of cell populations is observed in fibroblast cultures from donors of all ages, representing a terminal differentiation lineage with a progenitor compartment with potentially mitotic fibroblasts and a functional compartment with postmitotic but metabolically active cells that can remain functional for many months, if not years (Bayreuther et al., 1988a, b; Bayreuther et al., 1992). In early-passage cultures established from human skin, three subtypes of progenitor fibroblasts can be distinguished which have been characterized at the morphological and biochemical level (Rodemann et al., 1989). Treatment with cytotoxic agents such as the alkylating agent Mitomycin C or ionizing radiation induces premature differentiation terminal to a postmitotic phenotype characterized by an increase in cell size with enlarged or multiple nuclei and increased synthesis of ECM proteins (Rodemann, 1989; Rodemann et al., 1991; Herskind et al., 2000). Thus radiation-induced differentiation of fibroblasts has been proposed as a cellular basis of radiation-induced fibrosis (Rodemann and Bamberg, 1995; Herskind et al., 1998).

Most studies on radiation-induced gene expression in fibroblasts have focused on the early time interval 1–24 h after irradiation, and identified genes involved in signaling, RNA and DNA synthesis, metabolism, DNA damage response and cell-cycle arrest (Khodarev et al., 2001; Ding et al., 2005; Kis et al., 2006; Zhou et al., 2006; Kalanxhi and Dahle, 2012). However, the differentiated phenotype is not expressed at such early time points. Rodningen et al. (2005) analyzed gene expression 2 and 24 h after irradiation of confluent cultures of skin fibroblasts with a single dose of 3.5 Gy or 2 h after a fractionated scheme of 3 × 3.5 Gy given at 24 h intervals (Rodningen et al., 2005). In addition to radiation-responsive genes, differentially regulated genes were involved in ECM remodeling, Wnt signaling, and IGF signaling (Rodningen et al., 2005), and a 13-gene signature for predicting radiotherapy patients individual risk of developing subcutaneous fibrosis was identified (Alsner et al., 2007; Andreassen et al., 2013). Tachiiri et al. (2006) studied gene expression at 1–72 h after doses in the range 0.5–50 Gy and found upregulation of two collagens (COL1A1 and COL5A1) in the late phase after giving a dose of 5 Gy (Tachiiri et al., 2006). A more recent study investigated radiation-induced senescence and found a strong increase in expression of SA-βGal positive cells 72–120 h after high single doses of 15–20 Gy with pathway analysis showing differential regulation of cell cycle-related genes (Marthandan et al., 2016). However, these studies did not consider other functional changes associated with radiation-induced differentiation of fibroblast.

The purpose of the present work was to study gene expression profiles in relation to radiation-induced phenotypic changes of human skin fibroblasts relevant for radiogenic fibrosis. Early-passage fibroblasts were irradiated in sparse cultures in order to allow expression of the differentiated phenotype, and differential gene expression was analyzed 2–5 days after irradiation. Furthermore, the expression profiles of the previously published irradiation protocol for predictive testing and the present protocol were compared.

## Materials and Methods

### Cell Culture

Three strains of primary human skin fibroblasts, GS3, GS4, and GS5, were a gift from Dr. J. H. Peacock, Institute of Cancer Research, Sutton, United Kingdom. The completely anonymized strains were established prior to the Human Tissue Act 2004 by outgrowth of explants taken from surplus tissue obtained during surgical reduction mammoplasty [breast reduction (Carlomagno et al., 2000)] on healthy donors younger than 40 years of age (J. H. Peacock, personal communication). The GS (“gold standard”) fibroblast strains (Kote-Jarai et al., 2004) were made available to the EU BIOMED 2 Concerted Action “The development of predictive tests of normal tissue response to radiotherapy” (European_Commission, 1999). For the present experiments, the cultures were grown in AmnioMax C-100 Medium supplemented with 7.5% AmnioMax supplement (Gibco/Invitrogen, Darmstadt, Germany) and 7.5% fetal bovine serum (FBS, South American origin; Biochrom, Berlin, Germany; HyClone, Fisher Scientific GmbH, Schwerte, Germany), 2 mM glutamine, penicillin, and streptomycin at 37°C under 7% CO_2_. In the Mannheim (MA) protocol, early-passage cells were seeded in sparse mass culture in T75 flasks (0.5 × 10^6^ cells/flask; Falcon) and irradiated in exponential growth phase with 4 Gy of 6 MV X-rays on the following day (day 0). For determining changes in cell densities after irradiation, the cells were seeded on microscope slides at the same density (6.67 × 10^3^/cm^2^), fixed at different time points and stained with Coomassie blue and Giemsa as previously described (Herskind et al., 1998). The cells were photographed and relative changes in cell numbers were determined by scoring the number of cells per area.

The design of the microarray experiments and quantitative real-time reverse transcription (RT-) PCR (qPCR) experiments is shown in Table 1. Two technical replicate experiments (#1 and #2) were performed with GS4 fibroblasts. RNA was isolated from T75 flasks on day 1 (0 Gy) and day 2, 3, and 5 (4 Gy), or day 5 (2 × 4 Gy), yielding approximately the same number of cells per unit area in unirradiated and irradiated flasks (MA protocol). Furthermore, the protocol used by Overgaard’s group in Aarhus (AR protocol) was run in parallel in these experiments. In this protocol, confluent cultures were irradiated with three fractions of 4 Gy of 6 MV X-rays (equivalent to 3.5 Gy of orthovolt X-rays) given on day 0, 1, and 2, and RNA was isolated 2 h after the last fraction (Rodningen et al., 2008; Andreassen et al., 2013). To verify differentially regulated genes and pathways common to all three fibroblast strains, experiments using the MA protocol with RNA isolation on day 3 after irradiation were performed (experiments #3–#5). Additional, independent experiments to test the kinetics and validate the microarray data for selected genes were performed with RNA isolation at 24 h intervals on day 1–6 after irradiation and determined gene expression by qPCR. Supplementary Experiments for qPCR validation and protein detection were performed with isolation of RNA and protein at selected time points as indicated.

**TABLE 1:** Experimental design of microarray and qPCR experiments.

*Fib. strain*	*Cult. seed.*	*Irrad. dose*	*Day (d) on which RNA was isolated*						*Protocol and type of experiment*
GS4	Expon.	0 Gy	**d1**						MA protocol
GS4	Expon.	4 Gy		**d2**	**d3**		**d5**		Microarrays; exp. #1–2
GS4	Expon.	2×4 Gy			4 Gy		**d5**		Microarrays; exp. #1–2
GS4	Confl.	0 Gy	0 Gy	0 Gy + **2h**					AR protocol
GS4	Confl.	4 Gy	4 Gy	4 Gy + **2 h**					Microarrays; exp. #1–2
GS3-5	Expon.	0 Gy	**d1**						MA protocol
GS3-5	Expon.	4 Gy			**d3**				Microarrays; exp. #3–5
GS3-5	Expon.	0 Gy	d1						MA protocol
GS3-5	Expon.	4 Gy	**d1**	**d2**	**d3**	**d4**	**d5**	**d6**	qPCR; kinetics/validation
GS3-5	Expon.	0 Gy	**d1**	**d2**	**d3**		**d5**		MA protocol suppl. exp.
GS3-5	Expon.	4 Gy		**d2**	**d3**		**d5**		qPCR; WB suppl. kinet./valid.
***→→→→***		∣***→→→***	∣***→→→***	∣***→→→***	∣***→→→***	∣***→→→***	∣***→→→***	∣***→→***→∣	Post-IR day (d) after first dose
−1		0	1	2	3	4	5	6	Post-IR day (d) after first dose

*Columns: ‘Fib. Strain,’ individual strains: GS3, GS4, GS5; ‘Cult. seed’, culture seeded, expon.(exponential growth phase), confl. (confluent cultures); ‘Irrad. dose’, irradiation dose; ‘Isolate RNA’, post irradiation time d1–d6 (day 1–6). MA, Mannheim protocol; AR, Aarhus protocol. Cells were irradiated on day 0, and also on d1 and d2 for the AR protocol. The time line is shown at the bottom. WB: Western blots.*

### Fibroblast Phenotype

Sparse mass cultures were seeded in chamber slides and irradiated with 1 × 4 Gy as described above. On day 1–5, the cells were fixed using 3.7% paraformaldehyde in PBS followed by staining with Coomassie blue/Giemsa as described (Herskind et al., 1998). Cell morphology was visualized in bright-field microscopic images using 10–20× objectives. For quantification of the size distributions, the areas of 300–600 individual cells for each condition were determined in additional repeat experiments using Image J software (Schneider et al., 2012). For quantitative changes in cell numbers, 100 cells were seeded per well in six-well plates, irradiated and fixed on day 1, 3, and 6 as described above. Cell numbers were counted in six wells per plate under a microscope. The colony formation assay (CFA) was used to determine changes in fibroblast phenotype (differentiation state, surviving fraction). Cells were seeded in triplicate T75 flasks at 100 cells/flask for cytomorphological scoring, and at 300–4,000 cells/flask for scoring colonies. After 11 days incubation, the cells were fixed and stained, and the L:E ratio between colonies (min. 50 cells) in late (L) and early (E) differentiation state was determined by cytomorphological scoring as previously described (Herskind et al., 1998; Herskind and Rodemann, 2000). The yields of postmitotic differentiated fibrocytes (PMF) and clones with less than 50 cells (large clusters: 11–49 cells; small clusters: 2–10 cells) were determined by cytomorphological scoring of single cells with no nearest neighbors within 2.5 mm in CFA seeded at low cell density (100 cells per T75 flask) as described (Herskind et al., 2000).

### Immunocytochemistry

Cells were seeded in chamber slides (BD Biosciences, Heidelberg, Germany) at a density of 6 × 10^3^ cells/cm^2^, the day before irradiation with 4 Gy. 1–5 days after irradiation, cells were fixed in methanol, washed and blocked with 1% bovine serum albumin in phosphate-buffered saline with 0.2% Triton-X100 (PBST). Incubation with 1:300 primary anti-α-smooth muscle actin (α-sma, ACTA2) mouse monoclonal antibody (sc-32251, Santa Cruz Inc., Heidelberg) in PBST was done for 1h. After washing three times with PBST, the slides were incubated with secondary FITC-conjugated goat anti-mouse IgG (1:20000) in PBST for 1h in the dark, washed three times and covered. The cells were photographed at 400× magnification with an inverted fluorescence microscope (Axio Observer.Z1, Carl Zeiss Microscopy Deutschland GmbH, Oberkochen, Germany).

### Immunoblotting

Protein lysates were prepared on the indicated days. Cells were lysed on ice by incubation with ice-cold RIPA lysis buffer, including the Complete Protease Inhibitor Cocktail (Roche, Mannheim, Germany). The lysates were centrifuged at 12,000 × *g* for 10 min at 4°C and the protein concentration was determined by the Bradford method. Cell lysates (20 μg of total protein) were mixed with equal volumes of 2 × Laemmli sample buffer with 5% β-mercaptoethanol and 1 mM dithiothreitol, denatured at 97°C for 5 min and separated by electrophoresis in 12% Bis–Tris acrylamide gels. The proteins were electroblotted onto Amersham Protran 0.2 μm nitrocellulose membranes (GE Healthcare, Freiburg, Germany) and probed with the following primary antibodies: anti-CCNB1 (ab32053) and anti-PTX3 (ab190838) from Abcam (Berlin, Germany), anti-RPA1 (#2267) and anti-NBN (#2267) from Cell Signaling Inc. (Frankfurt a.M., Germany), anti-α-smooth muscle actin (α-sma, ACTA2; sc-32251) from Santa Cruz Inc. (Heidelberg, Germany), and anti-ACTB (A5441) from Sigma-Aldrich/Merck (Darmstadt, Germany). After washing, the membranes were incubated with horseradish peroxidase-conjugated secondary antibody (P0447, P0448; Agilent/Dako, Hamburg, Germany) for 1 h at room temperature, washed, processed with a Western Lightning Plus ECL kit (PerkinElmer, Hamburg, Germany) and fluorescence detected with a Fusion FX7/SL Advance imaging system (Vilber Lourmat, Eberhardzell, Germany). Band intensities were quantified with Image J software (Schneider et al., 2012), corrected for the intensity of the loading control ACTB, and normalized to the values on day 1 of the unirradiated samples.

### RNA

Total RNA was prepared using Trizol (Gibco) followed by additional purification using the RNeasy Mini Kit (Qiagen). RNA was tested by capillary electrophoresis on an Agilent 2100 bioanalyzer (Agilent) and high quality was confirmed. The RNA samples from exp. #1 and exp. #2 were stored and processed separately for each experiment but biostatistical analysis was performed together. RNA samples from exp. #3–#5 were stored, processed and analyzed together. Biotinylated antisense cRNA was prepared according to the Affymetrix standard labeling protocol with the GeneChip^®^ WT Plus Reagent Kit and the GeneChip^®^ Hybridization, Wash and Stain Kit (both from Affymetrix Inc., Santa Clara, CA, United States). RNA samples for qPCR were stored for each experimental series (15 genes in the main text, 9 and 3 genes in Supplementary Experiment) and the assays were performed over a short period of time.

### Microarrays

Gene expression profiling was performed using Human Genome U133 Plus 2.0 microarrays (Affymetrix Inc., Santa Clara, CA, United States). Hybridization was performed in a GeneChip Hybridization oven 640, then dyed in the GeneChip Fluidics Station 450 and thereafter scanned with a GeneChip Scanner 3000. All of the equipment used was from Affymetrix UK Ltd. (High Wycombe, United Kingdom).

### Irradiation and Dosimetry

Irradiation was performed with 6 MV X-rays from a clinical radiotherapy machine (Elekta Synergy, Elekta Oncology Systems, Crawley, United Kingdom) with a dose rate of 6 Gy/min. Dose build-up above the cells was equivalent to 15 mm water depth and 8 cm water-equivalent backscatter material was added below the flasks. Dosimetry was performed as part of the daily quality check for radiation therapy.

### Bioinformatics

A Custom CDF Version 24 with ENTREZ based gene definitions was used to annotate the arrays (Dai et al., 2005). All samples assigned to a gene were summarized using the RMA method (Irizarry et al., 2003). The raw fluorescence intensity values were normalized by applying quantile normalization and RMA background correction for exp. #1 and #2 combined, and for exp. #3–#5 combined. In order to minimize variations between independent experiments, batch correction was performed using the batch normalization process of the JMP Genomics Software version 7 (SAS Institute, Cary, NC, United States). In brief, a *K*-Means clustering is applied to group batch profiles into clusters and the batch normalization is performed by correcting the within-cluster mean profile for each cluster. The resulting expression levels were used to determine the log2 values of fold change [log2(FC)], in each experiment by subtracting the expression level in unirradiated fibroblasts from that of irradiated cells at a given time point. The independent replicates for GS4 (exp. #1 and #2) showed a high degree of correlation (Supplementary Figures 1A–E). Therefore, data were filtered for mean log2(FC) > 2 or <−2 and 95% confidence intervals were determined from the variance of the difference of individual replicates and their mean value (within the filtered gene sets, which was 48 ± 11% larger than the variance for the complete gene set). Notably, all individual replicate data points in the filtered gene sets (2*n* = 3670) showed absolute log2(FC) values > 1.4 corresponding to larger than 2.6-fold changes. The differential expression in a completely independent experiment with GS4 (exp. #3) showed excellent correlation (*R*^2^ = 0.95) with the mean values of the filtered genes from experiments #1 and #2 although, numerically, log2(FC) values were 15% smaller overall (Supplementary Figure 1F). Importantly, however, all of the filtered genes were regulated in the same direction in all three experiments with none of the genes showing changes in opposite directions. Out of 113 up- and 232 down-regulated genes identified in the first two experiments, 97% showed at least twofold changes. Only five genes in each group showed log2(FC) values in experiment #3 in the range 0.6–1.0 (corresponding to 1.5–2-fold changes), while a single uncharacterized LOC gene showed log2(FC) = 0.42 (corresponding to 1.34-fold up-regulation). The strong agreement between replicate experiments suggests that false discoveries of up- or down-regulated genes are extremely unlikely within the filtered gene sets.

The same method of filtering and determination of the variance of the filtered gene set around mean values for each gene was used to analyze experiments #3–#5, yielding mean values and confidence intervals for differentially expressed genes in fibroblast strains (GS4, GS3, and GS5) from three individual donors. In this case, absolute log2(FC) values were larger than 0.66 corresponding to larger than 1.5-fold changes for all individual data points (*n* = 966) within the filtered gene set (122 up- and 200 down-regulated genes). In this case, the variance between strains was 6.5-fold larger for the filtered genes than for the whole gene set, implying that quantitative variations between fibroblast strains from individual donors were larger than the technical variation between experiments. Nevertheless, the direction of the differential regulation was the same in all strains for all 322 filtered genes.

Gene Set Enrichment Analysis (GSEA) was used to determine whether defined lists (or sets) of genes exhibit a statistically significant bias in their distribution within a ranked gene list using *ReactomePA* and ClusterProfiler in the software package R (Yu and He, 2016). The gene list was ranked according to the log2(FC) values of each gene for irradiated versus unirradiated cultures. Curated pathways over- or under-represented in differentially expressed genes were identified by normalized enrichment scores (NES). Pathways with *p*-values < 0.05 after adjustment for multiple testing were considered significant.

The raw and normalized data are deposited in the Gene Expression Omnibus (GEO) database^1^ (accession number GSE147733).

### Quantitative Real-Time RT-PCR (qPCR)

Validation of selected genes was done by qPCR using RNA isolated from independent repeat experiments performed once for each strain 1 year after the original experiments. qPCR was performed with SYBR-green using Gene Globe primer and detection system according to the manufacturer’s protocol (Qiagen, Hilden, Germany). The fold induction was calculated by the ΔΔCt method using *ACTB* as control gene. Mean values and standard errors for the three strains are shown. *ACTB* was chosen as control gene based on pilot experiments showing no significant change in *C*t values with time after irradiation (day 0–6, *P* = 0.77, *n* = 20; ANOVA) for the three fibroblast strains. This was corroborated when analyzing all *ACTB* expression data from the qPCR validation experiments for the 15 genes although minor but significant deviations (*P* < 0.001, *n* = 105; ANOVA) were observed for day 2 and day 3 (Supplementary Figure 2).

### Statistics

Mean values and standard deviations, standard errors, or 95% confidence intervals are shown as indicated in the graphs. Statistical significance was tested by student’s *t*-test or by ANOVA. JMP statistical discovery software v.14 (Böblingen, Germany) and Excel (MS Office 2016) were used.

## Results

### Radiation-Induced Change of Fibroblast Phenotype

**FIGURE 1:**
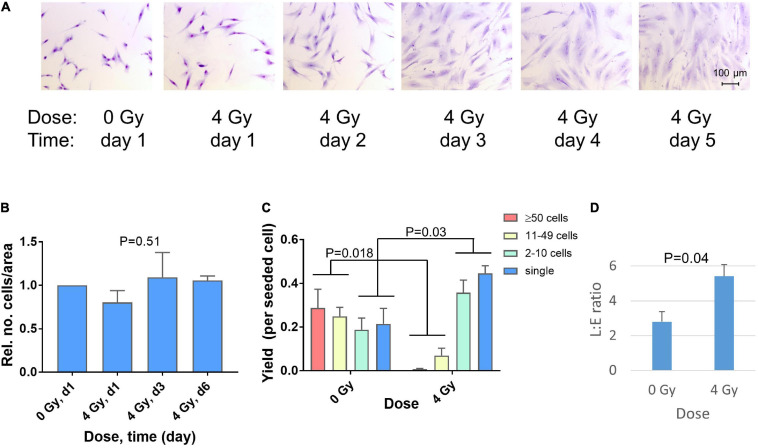
Changes in cell morphology 1–5 days after irradiation of GS4 in microscope chamber slides with a dose of 4 Gy **(A)**. Relative numbers of cells on day 1, 3, and 6 after irradiation of GS3, GS4, and GS5 in six-well plates with 4 Gy. For each fibroblast strain, the total number of cells in all six wells of the plate was scored. *P* = 0.51 (ANOVA, *N* = 12, *n* = 3 per group) **(B)**. The yield of clones per seeded cell in the colony formation assay (T75 flasks) with GS3, GS4, and GS5, irradiated at low cell density after doses of 0 Gy (sham) and 4 Gy. Cytomorphological assay (clonal culture at low cell density), clones sizes: ≥50 cells (colonies), 11–49 cells (large clusters), 2–10 cells (small clusters), single cells (postmitotic). *P*-values for changes in colonies + large clusters and small clusters + single cells are shown (unpaired *t*-test, *N* = 6) **(C)**. Ratio of colony-forming fibroblasts in late (L) and early (E) differentiation state in the colony formation assay for GS3, GS4, and GS5 irradiated with 0 Gy (sham) or 4 Gy. *P*-value for unpaired *t*-test (*N* = 6) **(D)**. Mean values and standard errors (*n* = 3) for the three strains are shown in **(B–D)**.

A marked change in fibroblast morphology was observed in micrographs after irradiation of fibroblasts in sparse cultures. On day one after irradiation, no change was visible. However, beginning on day 2, the cell size and morphology changed from typical spindle-like shape to large extended cells, representing radiation-induced premature differentiation (Figure 1A). Although the density of the cultures appeared to increase, this was largely due to the increase in cell size. Preliminary image analysis indicated that the major increase in cell size occurred between day 1 and 3 (not shown) and this was validated by analyzing the cell size distributions in three independent repeat experiments for day 1 and day 3 (Supplementary Figure 3). A shift in the frequency of cells with small areas toward larger areas (approximately >1,500 μm^2^) was observed in irradiated cultures. Thus the median area increased significantly (*p* = 0.02; *N* = 3, paired *t*-test) from 934 ± 131 μm^2^ (0 Gy, day 1) to 2,161 ± 46 μm^2^ (4 Gy, day 3). Additional experiments with GS3, GS4 and GS5 in six-well plates were performed to count the number of cells fixed on day 1, 3, and 6. This confirmed that the number of cells per unit area did not change significantly after irradiation (*P* = 0.51, *N* = 12, *n* = 3 per group), indicating permanent cell-cycle arrest of most cells after this dose (Figure 1B).

Clonogenic survival showed surviving fractions (SF) in the range 1.21–6.7% after 4 Gy. This dose was used because it was equivalent to the fraction size applied in postmastectomy patients in which fibrosis after radiotherapy had previously been studied (Johansen et al., 1996; Herskind et al., 1998; Alsner et al., 2007). Microscopic scoring of clone sizes showed a radiation-induced decrease in the number of colonies and clones with 11–49 cells after irradiation (*P* = 0.018, *N* = 6) which was approximately balanced by a significant increase (*P* = 0.03, *N* = 6) in the number of small clones with 2–10 cells and single post-mitotic cells (Figure 1C). Thus inactivated clonogenic cells were either arrested permanently or able to undergo max. 1–3 cell divisions in the time that undamaged cells would form colonies. The surviving colonies showed an approximately twofold increase in the L:E ratio between the number of colonies in late and early different state (geometric mean 2.07, range 1.55–2.76; *P* = 0.04, *N* = 6) implicating a shift in fibroblast differentiation after irradiation (Figure 1D). Taken together, these results show the radiation-induced phenotype of premature terminal differentiation developing 2–5 days after irradiation of fibroblasts in sparse cultures *in vitro* (i.e., after DNA repair and cell recovery are assumed to have been completed).

### Changes in Gene Expression Profile After Irradiation

**FIGURE 2:**
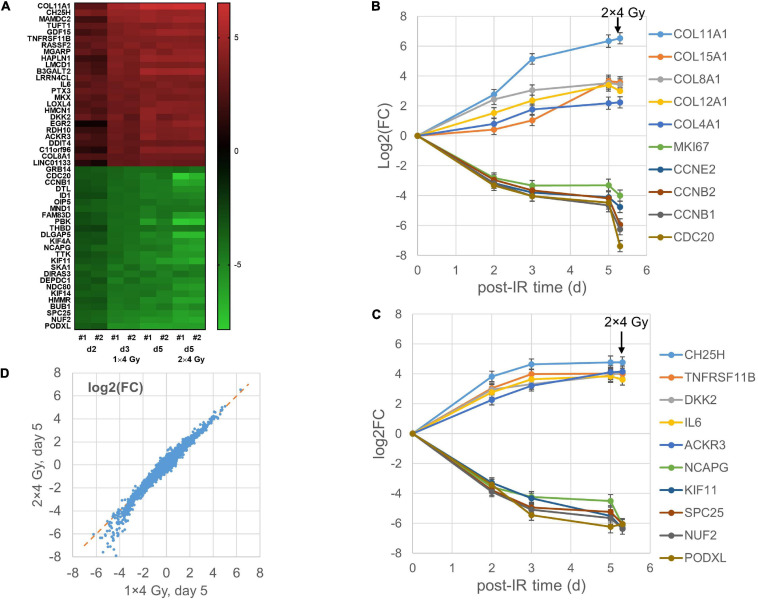
Heat map of top 25 up- and down-regulated genes for the MA protocol in independent replicate microarray experiments #1–2 **(A)**. Examples of log2 fold changes [log2(FC)] in expression of differentially regulated genes 2–5 days after a single dose of 4 Gy or 5 days after the first of two fractions of 4 Gy; mean values and error bars (95% confidence intervals) from the two experiments are shown **(B,C)**. Correlation between mean log2(FC) values after 2 × 4 Gy and 1 × 4 Gy **(D)** with RNA isolation on day 5 after the first dose.

Differential gene expression after the single-dose (MA) protocol (1 × 4 Gy, exponential growth) was studied for GS4 cells on day 2, 3, and 5 after irradiation. A parallel flask was given a second fraction of 4 Gy on day 3 after the first irradiation and RNA was isolated on day 5 (i.e., 2 days after the second fraction). Two independent replicate experiments were performed showing a high degree of correlation (*R*^2^ = 0.81–0.91; *n* = 20,422 genes; Supplementary Figure 1). Filtered genes with mean log2(FC) > 2 or <−2 (i.e., at least fourfold up- or down-regulation) are included with 95% confidence intervals in a Supplementary Excel Table. All filtered genes showed changes in the same directions in both replicate experiments and were significant at the 95% confidence level. Heat maps of the top 25 up- and down-regulated genes [mean log2(FC)] on day 2, 3, and 5 after irradiation are shown in Figure 2A and mean values and 95% confidence intervals for the top 25 up- and down-regulated genes are listed in Supplementary Table 1. The kinetics of selected genes showed differences in the upregulation of different collagen genes (Figure 2B), either a continuous increase (*COL11A1* and *COL12A1*), leveling off on day 3–5 (*COL4A1* and *COL8A1*), or a late increase (*COL15A1*). By contrast, proliferation- and cell division-related genes were down-regulated in a broadly similar way (Figure 2B). Other genes showing stronger up- or down-regulation on day 2 tended to change less on day 3–5 (Figure 2C). The differential expression after giving two fractions of 4 Gy was highly correlated with that observed 5 days after giving a single fraction (Figure 2D) and less so with the earlier time points (*R*^2^ = 0.93 versus 0.88 and 0.76 for day 3 and 2, respectively; data not shown). Thus, the transcription signature for 2 × 4 Gy was more similar to the time after the first fraction (5 days) than the second (2 days). However, whereas upregulated genes showed little effect of the second fraction, some of the most down-regulated genes on day 5 seemed to be more strongly down-regulated after the second dose (Figures 2B–D).

**FIGURE 3:**
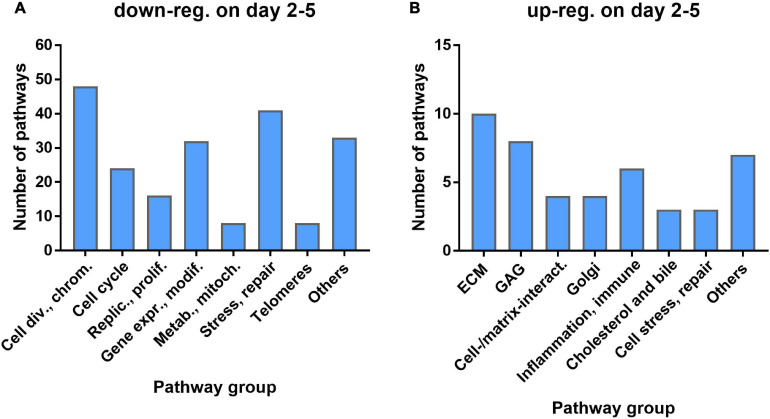
Number of pathways by functional group for pathways commonly down-regulated **(A)** or up-regulated **(B)** at all three time points.

GSEA was used to identify significant pathways with NES > 1.5 (over-represented, i.e., upregulated after irradiation) or <−1.5 (underrepresented, i.e., downregulated after irradiation). 268 pathways were downregulated at least at one of the time points d2, d3, or d5, with 216 (81%) downregulated at all three time points, while 106 pathways were upregulated at one or more of the three time points with 45 (42%) common to all three (Supplementary Figure 4). 40% of the 216 common down-regulated pathways were related to cell division, chromosome organization, or cell-cycle and replication/proliferation, with additional 18% related to gene expression and protein modification, and 19% to cell stress and DNA repair (Figure 3A). 49% of the 45 common up-regulated pathways were related to the ECM, GAG, or cell–cell and cell–matrix interactions (Figure 3B) with an additional 13% related to inflammation and immune reactions. The complete list of pathways is included in a Supplementary Excel Table.

### Influence of Irradiation Protocol on Gene Expression Pathways

**FIGURE 4:**
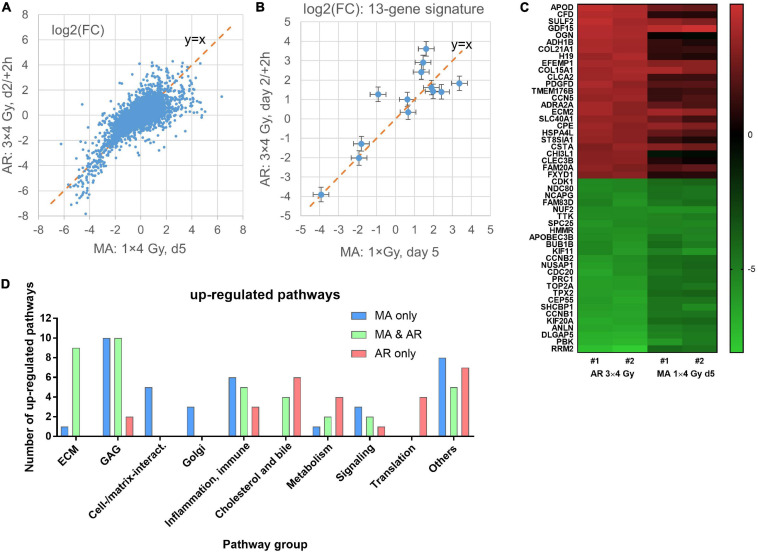
Correlation of mean log2 fold changes [log2(FC)] after irradiation of confluent cultures with 3 × 4 Gy (AR protocol) and irradiation of exponentially growing cultures with 1 × 4 Gy (MA protocol) in microarray experiments #1–2 **(A)**. Same for the 13 genes in the predictive signature for subcutaneous fibrosis (Alsner et al., 2007). Mean values and 95% confidence intervals for data from experiments #1–2 are shown **(B)**. Heat map of the top25 up- and down-regulated genes for irradiation with the AR protocol and the same genes on day 5 for the MA protocol; data from independent replicate experiments #1–2 **(C)**. Number of pathways by functional group for pathways that were significant for only one of the two or common to both protocols **(D)**.

Comparing the differential expression of the AR protocol showed the best correlation (*R*^2^ = 0.52) with MA day 5 (Figure 4A). Downregulated genes showed a higher degree of correlation but strongly down-regulated gene seemed to be more down-regulated in the AR than in the MA protocol. Upregulated genes tended to be less up-regulated in the AR protocol but considerable divergence was observed and this was also the case for the 13-gene predictive signature (Figure 4B). The top 25 up- and down-regulated genes for the AR protocol are listed in Supplementary Table 2 and shown as a heatmap together with day 5 for the MA protocol (Figure 4C). Downregulated genes, including several cell cycle- and cell division-related genes, showed overlap with the MA protocol (day 5) for nine genes. By contrast, only one of the top 25 up- regulated genes showed overlap (*GDF15*) although *COL15A1*, which was outside top 25 on day 5 of the MA protocol, was upregulated to a similar degree [log2(FC) = 3.65]. The complete list of differentially regulated genes is included in the Supplementary Excel Table. Pathway analysis showed 37 up-regulated pathways common to both protocol with 52% relating to the ECM or GAG, and 14% to inflammation and the immune system (Figure 4D). Additional 37 upregulated pathways were significant for MA d5 with 41% relating to GAG or cell–cell/cell–matrix interactions and only one pathway to ECM. By contrast, among the 27 pathways significantly upregulated for AR only, just 7% were related to GAG and none to ECM or cell–cell/cell–matrix interactions. Instead, 22% were related to cholesterol and bile acid/salts pathways, 15% to translation, and 15% to metabolism. Both protocols also upregulated pathways related to inflammation and immune reactions that were significant for only the MA and not the AR protocol or vice versa.

### Robustness of Radiation-Induced Pathways in Different Fibroblast Strains

**FIGURE 5:**
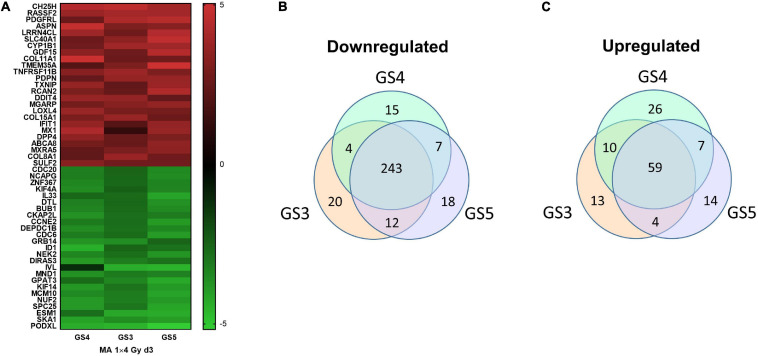
Heat map of top25 up- and downregulated genes in the three fibroblast strains (GS3-5) on day 3 in microarray experiments #3–5 **(A)**. Venn diagrams for pathways upregulated **(B)** or downregulated **(C)** on day 3 after a single dose of 4 Gy to the three fibroblast strains GS3, GS4, and GS5.

The robustness of the gene expression profile obtained with GS4 for d3 (MA protocol) was tested in a second series of microarray experiments including two further fibroblast strains, GS3 and GS5. The top25 mean log2(FC) upregulated genes for all three strains are shown as a heatmap (Figure 5A) and mean log2(FC) values and 95% confidence intervals in Supplementary Table 3. The complete list of filtered genes is included in the Supplementary Excel Table. qPCR validation of 27 genes is described below. Pathway analysis showed 320 downregulated pathways, 243 of which were common to all three strains (Figure 5B). The major groups were similar to the previous results for GS4 on day 2–5. 125 pathways were upregulated with 59 being common to all three strains (Figure 5C). The top 25 common pathways (Table 2) ranked by the mean NES for the three fibroblast strains confirmed the high fraction of ECM-related pathways but also included four pathways related to inflammation and immune reaction: Interferon alpha/beta signaling (R-HSA-909733) and three pathways involving the complement system. Further six inflammatory pathways with NES values in the range from 1.59 to 1.94 were outside top 25, including Interferon gamma signaling (R-HSA-877300) and Interleukin-20 family signaling (R-HSA-8854691). Thus 10/59 pathways (17%) upregulated on day 3 in all three strains represented early aspects of inflammatory signaling. The complete list of differentially regulated pathways is included in the Supplementary Excel Table.

**TABLE 2:** Top25 upregulated pathways overrepresented with NES > 1.5 and adjusted *p*-value < 0.05 in each of the three fibroblast strains (GS3, GS4, and GS5) on day 3 after irradiation with 1 × 4 Gy (MA protocol).

ID	Description	Gene set size	Mean NES	Adjusted *p*
R-HSA-1650814	Collagen biosynthesis and modifying enzymes	67	2.43	<0.0016
R-HSA-909733	Interferon alpha/beta signaling	62	2.43	<0.0016
R-HSA-1474244	Extracellular matrix organization	297	2.40	<0.0016
R-HSA-8948216	Collagen chain trimerization	44	2.40	<0.0016
R-HSA-1474290	Collagen formation	90	2.31	<0.0016
R-HSA-3000178	ECM proteoglycans	75	2.27	<0.0016
R-HSA-1566948	Elastic fiber formation	44	2.27	<0.0016
R-HSA-2022090	Assembly of collagen fibrils and other multimeric structures	61	2.27	<0.0016
R-HSA-1442490	Collagen degradation	63	2.24	<0.0016
R-HSA-166658	Complement cascade	51	2.20	<0.0016
R-HSA-2129379	Molecules associated with elastic fibers	37	2.19	<0.0016
R-HSA-381426	Regulation of Insulin-like Growth Factor (IGF) transport and uptake by Insulin-like Growth Factor Binding Proteins (IGFBPs)	120	2.19	<0.0016
R-HSA-977606	Regulation of Complement cascade	40	2.17	<0.0016
R-HSA-8957275	Post-translational protein phosphorylation	104	2.16	<0.0016
R-HSA-1474228	Degradation of the extracellular matrix	137	2.15	<0.0016
R-HSA-186797	Signaling by PDGF	58	2.09	<0.0016
R-HSA-194068	Bile acid and bile salt metabolism	43	2.04	<0.0049
R-HSA-216083	Integrin cell surface interactions	84	2.04	<0.0016
R-HSA-211976	Endogenous sterols	27	2.03	<0.0016
R-HSA-2173782	Binding and Uptake of Ligands by Scavenger Receptors	39	2.03	<0.0063
R-HSA-192105	Synthesis of bile acids and bile salts	34	2.01	<0.0038
R-HSA-2243919	Crosslinking of collagen fibrils	18	1.99	<0.0099
R-HSA-3000171	Non-integrin membrane-ECM interactions	58	1.99	<0.0115
R-HSA-2022857	Keratan sulfate degradation	13	1.98	<0.0027
R-HSA-166663	Initial triggering of complement	20	1.96	<0.0063

*Mean NES, mean value of normalized enrichments score for the three strains. ‘Adjusted p’ shows the largest adjusted p-values from separate pathway analyses for each of the three strains. NES values and adjusted p-values for each strain are given in the Supplementary Excel Table.*

### Expression Kinetics and qPCR Validation of 15 Selected Genes

**FIGURE 6:**
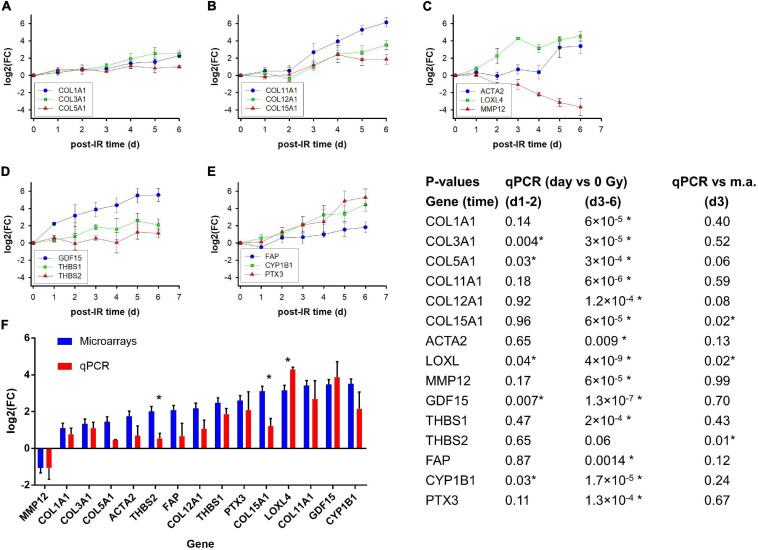
Kinetics of gene expression (fold induction determined with qPCR) of selected genes on day 1–6 after irradiation of GS3, GS4, and GS5 fibroblasts with a single dose of 4 Gy; mean values and standard errors of *n* = 3 independent experiments are shown **(A–E)**. Comparison of log2 fold changes [log2(FC)] for microarrays and qPCR on day 3 after a single dose of 4 Gy. The microarray and qPCR assays were performed on RNA from GS3, GS4, and GS5 irradiated in separate, independent experiments; Mean values and standard errors are shown **(F)**. The table shows *P*-values for log2(FC) of irradiated samples relative to unirradiated controls at early time points (day 1–2 together) and late time points (d3–d6 together) for the qPCR experiments (heteroscedastic *t*-test, *N* = 9 and 15, respectively) and for qPCR versus microarrays (m.a.) from experiments #3–5 for GS3, GS4, and GS5 (unpaired *t*-tests, *N* = 6). Asterisks indicates *P* < 0.05.

To study the expression kinetics of selected genes and validate the microarray results by qPCR, additional independent experiments were performed with irradiation of the three strains isolating RNA each day from day 1 to day 6 (Figures 6A–E). Genes were selected based on the filtered lists and their potential relevance for the extracellular matrix, differentiation and fibrosis. Ten genes were part of the filtered gene list from exp. #1–2 as well as exp. #3–5. *MMP12* and *ACTA2* were in the filtered gene list for exp. #1–2 (GS4 only), and although *COL1A1*, *COL3A1*, and *COL5A1*, were in neither of the filtered gene lists, they were included because they are the major fibrillary collagen genes. Several collagen genes (*COL11A1, COL15A1, COL12A1, COL5A1, COL3A1*, and *COL1A1*) and *LOXL4*, the product of which is involved in trimerization of collagen fibers, were upregulated to different levels. Notably, of the three collagen genes which were upregulated >twofold on microarrays although they did not pass the stringent filtering (∣log2(FC)∣ > 2), *COL1A1* and *COL3A1* were continuously upregulated up to day 6 while *COL5A1* reached a lower plateau on day 4. Furthermore, the *FAP* gene coding for a serine protease was moderately upregulated while *MMP12* coding for matrix metalloproteinase 12 was downregulated.

*GDF15* coding for a TGF-beta superfamily cytokine was strongly upregulated. *THBS1* coding for the signaling glycoprotein, thrombospondin 1, showed intermediate upregulation up to day 5 while *THBS2* showed a moderate response with a plateau on day 1–4. *CYP1B1* coding for the cytochrome P450 1B1 enzyme was strongly upregulated similar to the *PTX3* gene coding for a long pentraxin. Expression of *ACTA2* coding for the myofibroblast marker α-smooth muscle actin (α-sma) was only upregulated on day 5–6 after irradiation.

A comparison of the log2(FC) values for microarray and PCR analyses for the three strains on day 3 showed a reasonably good correlation, considering that the RNA was isolated from two individual series of experiments separated by approximately 9 months (Figure 6F). Some genes with moderate upregulation showed lower log2(FC) than for microarrays but only three were significant, two of which (THBS2, COL15A1) showed lower log2(FC) values (*P* = 0.01 and 0.02, respectively; unpaired *t*-test, *n* = 2 × 3 per gene) and one (LOXL) a higher value (*P* = 0.02). Overall, the correlation for all three strains yielded *R*^2^ = 0.50 which increased to *R*^2^ = 0.72 when GS3 was excluded. More detailed analysis (not shown) suggested that the GS3 qPCR experiment underestimated the log2(FC) of some genes, possibly owing to experimental factors. Nevertheless, the full kinetics including other time points (Figures 6A–E) supported the radiation-induced differential expression obtained from the microarray experiments.

### Additional Validation of Gene Expression at the RNA and Protein Levels

It may be argued that part of the time-dependent changes in gene expression may be related to culture conditions rather than irradiation. In order to test this, additional irradiation experiments were undertaken, including unirradiated controls at the time points on day 2, 3, and 5 in addition to day 1 used in the microarray experiments. Because unirradiated cultures would reach confluence and might undergo density arrest during the experiment, unirradiated cultures were seeded at lower densities to reach approximately the same cell density as the irradiated cultures at the time of RNA and protein isolation. mRNA expression of nine genes determined by qPCR are shown in Supplementary Figure 4. For some of the genes, notably the cyclins *CCNB1* and *CCNE2*, expression in unirradiated controls actually showed some decrease with time. This may be explained by a slowing-down of proliferation, possible due to serum depletion, but from previous experience, these cultures do not lose the capacity to proliferate after reseeding into new flask. In spite of the changes with time in unirradiated cultures, the effect of irradiation was statistically significant at *P* < 0.05 in 6/9 genes on day 3 and in 7/9 genes on day 5, with strong trends (*P* < 0.09) in the remaining genes. The effect of irradiation was supported by *K*-means clustering analysis which separated day 3 and 5 of all irradiated samples from unirradiated samples and irradiated samples day 2 (not shown). Western blotting confirmed down-regulation of CCNB1 in unirradiated samples but downregulation was stronger after irradiation. Furthermore, PTX3 and α-sma were upregulated significantly on day 3–5 in irradiated compared to unirradiated cultures (Supplementary Figures 5, 6A,B). Immunofluorescence staining showed that upregulation of α-sma did not occur uniformly in all cells but showed contiguous stress fiber-like patterns covering small clusters of cells (Supplementary Figure 6C), suggesting the formation of small contractile cell clusters.

Pathway analysis showed downregulation of several stress and repair pathways after irradiation (MA protocol; Figure 3A). This may appear counter-intuitive if it is assumed that cells are arrested because of incomplete repair. However, permanent cell-cycle arrest may result from unrepaired or misrepaired DNA double-strand breaks (DSBs) leading to formation of micronuclei or dicentric chromosomes. In order to confirm downregulation of repair-related genes, we identified *RPA1*, *NBN*, and *POLD3* as the most frequent genes in the top 3 of leading core genes in 41 down-regulated stress and repair pathways. These genes are involved in different repair pathways such as translesion synthesis (*RPA1* and *POLD3*), and DNA damage response and telomere maintenance (*NBN*). Irradiation experiments were performed with isolation of RNA and protein from irradiated and unirradiated cultures for early and intermediate time points (4–72 h). qPCR showed significant down-regulation of these genes (*P* = 0.0002–0.02) at 24–72 h relative to unirradiated fibroblasts at the same time points, and this was further validated at the protein level for RPA1 and NBN in Western blots (Supplementary Figure 7).

## Discussion

In the present work, genes and pathways that are differentially expressed during phenotypic changes following irradiation of primary skin fibroblasts *in vitro* were identified. The radiation-induced phenotype was characterized by inactivation of clonogenicity accompanied by a gradual increase in cell size and change in morphology typical of radiation-induced differentiation of proliferating fibroblasts to postmitotic fibrocytes (Bayreuther et al., 1988b; Rodemann et al., 1991; Herskind et al., 2000). Furthermore, surviving colony-forming progenitor fibroblasts showed a two–threefold increase in the L:E ratio indicating a shift in the differentiation state after irradiation as previously described (Herskind and Rodemann, 2000).

The main result is the overall change in down-regulated genes and pathways related to proliferation and the up-regulation of genes and pathways related to the extracellular matrix and inflammation. A previous study on the IMR90 fibroblast strain identified 1,381 genes with more than twofold down-regulation genes after replicative senescence or radiation-induced arrest 5 days after a single dose of 5 Gy, while 660 genes were more than twofold up-regulated (Lackner et al., 2014). The overlap between the two types of arrest was smaller for up-regulated (93/660 = 14%) than for down-regulated genes (298/1381 = 22%). The ratio of the number of down- to up-regulated genes after irradiation was 1.77:1 (530:299 genes), compared with 3.5:1 for more than fourfold upregulated genes in GS4 on day 5 (256:74 genes) in the present study.

Functionally, the downregulated genes were dominated by genes related to cell-cycle progression and mitosis, consistent with the phenotypic loss of proliferation. Genes such as *MKI67*, *CCNB1/2* and *CCNE2*, and *CDC20*, showed similarly strong (approximately eightfold) downregulation on day 2 leveling off on day 3–5. The high mean fold downregulation (10–50-fold) is a clear indicator of efficient shut-down of cell-cycle progression in the vast majority of the cells. Among the most downregulated genes, *PODXL* (podocalyxin-like) and *ID1* (inhibitor of DNA binding 1) have been associated with migration and epithelial-mesenchymal transition (EMT) (Scharpfenecker et al., 2009; Frose et al., 2018; Wong et al., 2019). *ID1* was originally identified as being repressed in senescent fibroblasts where its expression represses the expression of the endogenous cyclin-dependent kinase inhibitor p16/CDKN2A (Hara et al., 1994; Zheng et al., 2004). A number of the genes down-regulated in the present study (e.g., *MKI67, CCNB1*, and *ID3*) were commonly down-regulated in replicative senescence and senescence induced by a single high dose of 20 Gy (Marthandan et al., 2016).

Pathway analysis showed 40% of all down-regulated pathway being related to cell division/chromosomes, cell cycle, and replication/proliferation, and almost 20% related to stress/repair. Pathways belonging to the former group were previously found for replicative senescence (Lackner et al., 2014), while another study found repair pathways to be down-regulated during senescence 5 days after a high single dose of 20 Gy (Marthandan et al., 2016). However, not all proliferation-related genes are down-regulated. Thus, early >2-fold up-regulation of the anti-proliferative BTG2 gene 1–24 h was shown in fibroblasts after a single dose of 5 Gy (Tachiiri et al., 2006). In the present study, 2.5–3.8-fold upregulation [log2(FC) = 1.34–1.97] of BTG2 was observed in GS4 in the MA protocol, increasing from day 2 to day 5 but, in the AR protocol, 5.9-fold upregulation [log2(FC) = 2.55 ± 0.34] occurred 2 h after the last fraction (early after the last fraction but 2 days after the first). In the MA protocol, upregulation in the three fibroblast strains was 2.6-fold [mean log2(FC) = 1.36 ± 0.92] on day 3, suggesting that upregulation is not restricted to early time points.

Several ECM related genes and pathways were upregulated after irradiation with the MA protocol. Tachiiri et al. (2006) found late upregulation of COL1A1, COL5A1, and IGFBP5 48–72 h after 5 Gy, which were also upregulated in the present study although at lower levels [log2(FC) = 1.10 − 1.45] than the filtering criteria [log2(FC) > 2]. By contrast, collagen and thrombospondin genes in ECM receptor pathways, which were upregulated in our study, were down-regulated after senescence observed five days after a dose of 20 Gy in the study by Marthandan et al. (2016). These previous findings support the view that the up-regulation of ECM-related genes and pathways 2–5 days after irradiation of exponentially growing fibroblasts in the present study (MA protocol) represent a transcriptional signature for progression of progenitor fibroblast to functional, prematurely differentiated cells which can be metabolically active for several months or even years (Bayreuther et al., 1988b) rather than senescence as a precursor to cell death.

Our aim was to test the hypothesis that radiation-induced differentiation can be related to changes in gene expression pathways after irradiation of fibroblast strains *in vitro* and to provide a framework for future studies on potential mechanisms. In the following, some individual genes that are potentially interesting for further studies are discussed and briefly reviewed. Complete lists for data mining are included as Supplementary Material.

### Genes Related to the Extracellular Matrix

Several genes coding for collagens as a major component of the extracellular matrix (ECM) were upregulated after irradiation. Fibrillar collagens (Exposito et al., 2010) such as *COL1A1*, coding for the most abundant collagen (type I), and *COL3A1* coding for collagen type III which confers strength to the fibrils, showed five–sixfold upregulation over 5–6 days (qPCR) although they were not among the Top25 genes in microarrays. *COL5A1* coding for type V which regulates fibril thickness (Sun et al., 2011), showed twofold upregulation but *COL11A1* coding for collagen type XI which is frequently found in cartilage (Blaschke et al., 2000) was in Top25 at all time points and reached 80-fold upregulation in qPCR. Furthermore, *COL12A1* and *COL15A1* coding for type XII and XV fibril-associated collagens with interrupted triple helices (FACIT) (Ivanova and Krivchenko, 2014) were upregulated 4–12-fold. *LOXL4* coding for lysyl oxidase homolog 4, which regulates collagen fibril organization (Herchenhan et al., 2015), was upregulated up to 20-fold in qPCR. The differential upregulation of these genes strongly suggests that irradiation not only increases the production but also leads to a change in the properties of collagen fibers. Thus it is tempting to speculate that increased expression of collagen types XI, XII, and XV, may contribute to tissue hardening and rigidity in subcutaneous fibrosis. In addition, microarray analysis showed eightfold upregulation of *COL8A1* coding for non-fibrillar short-chain collagen type VIII which promotes remodeling and fibrosis (Skrbic et al., 2015), and fourfold upregulation of *COL4A1* coding for type IV which is essential to the sheet-like structure of basement membranes (Jayadev and Sherwood, 2017), both of which appeared to reach a plateau 3–5 days after irradiation. Finally, expression of *ACTA2* was upregulated with delayed kinetics on day 5–6. Its product, α-smooth muscle actin (α-sma, ACTA2), is a marker for myofibroblast differentiation and was observed in single cells or small clusters of adjacent cells separated by non-expressing cells. We speculate if this may represent formation of contractile cell clusters. Thus the irradiated cultures showed essential hallmarks of radiation-induced, premature terminal differentiation of mitotic progenitor fibroblasts to post-mitotic functional cells distinct from the senescent state (Bayreuther et al., 1988b; Rodemann et al., 1989, 1991; Herskind and Rodemann, 2000; Herskind et al., 2000).

Although downregulation of matrix metalloproteinases (MMPs) might have been anticipated to contribute to enhanced ECM deposition, most MMPs showed little or only moderate modulation in the three strains on day 3. *MMP12*, coding for macrophage metalloelastase was downregulated twofold on day 3 but increased to eightfold downregulation on day 6 in qPCR, while *MMP10* coding for stromelysin 2 was downregulated fourfold on day 3–5 in microarray analysis of GS4 which was confirmed for day 3 in all three strains. By contrast, *FAP* coding for a prolyl endopeptidase associated with tissue remodeling and fibrosis (Hamson et al., 2014) was upregulated from day 2, reaching fourfold on day 5–6. The related gene, *DPP4*, coding for dipeptidylpeptidase-IV was upregulated 6–11-fold on day 3 in the three strains and has been associated with organ fibrosis and systemic sclerosis (Min et al., 2014; Soare et al., 2020). *MXRA5* coding for matrix-remodeling-associated protein 5 (adlican), was upregulated on day three in microarray analysis in all three strains and has been described as anti-inflammatory and anti-fibrotic (Poveda et al., 2017). Taken together, these findings strongly implicated increased ECM deposition and remodeling rather than reduced collagen degradation in the fibrogenic process after irradiation. This was supported by pathway analysis showing collagen degradation among the top-10 upregulated pathways common to the three strains on day 3. Thus the balance between pro-fibrotic deposition of structurally modified ECM molecules and degradation as part of ECM remodeling mechanisms may determine the development of clinical fibrosis.

### Signals Leading to Phenotypic Changes

The signals initiating the gene expression program associated with radiation-induced differentiation and phenotypic changes are poorly understood. Transforming growth factor-β1 is considered a master switch in the development of fibrosis (Martin et al., 2000) but *TGFB1* was not among the radiation-induced upregulated genes in the present study. Latent TGF-β1 is stored in the ECM (and in blood platelet) and can be released by various agents such as including reactive oxygen species, reduced pH, and proteases such as plasmin and thrombospondin (Barcellos-Hoff and Dix, 1996; Ehrhart et al., 1997; Annes et al., 2003). However, in a previous study of fibroblasts *in vitro*, the total amount of active TGF-β1 released per flask released over a 24 h period from fibroblast cultures irradiated with 4 Gy was not significantly increased relative to unirradiated controls (Herskind and Rodemann, 2000). Nevertheless, the present study showed 3.5–6-fold upregulation of *THBS1* on day 2–6 and 1.5–3-fold for *THBS2*. Since the latter can antagonize TGF-β1 activation by THBS1 (Murphy-Ullrich, 2019) this provides a potential mechanism for a tightly regulated release of active TGF-β1 stored in the ECM or in blood platelets *in vivo*.

Perhaps a more important signal for the differentiation program leading to phenotypic changes is suggested by the early, strong upregulation of *GDF15* coding for the TGF-β1 family protein growth/differentiation factor 15 in the MA irradiation protocol and which was also upregulated in the AR protocol. GDF15 (also known as macrophage inhibitory cytokine-1, MIC-1) is upregulated by various stresses and has been associated with inflammation, cancer, and cardiovascular disease (Emmerson et al., 2018; Zhang et al., 2018; Kalli et al., 2019; Patel et al., 2019). Although it has been reported to signal via TGFβR2 and the downstream SMAD pathway, more recent studies implicated binding to a GFRAL/RET heterodimer with signaling to ERK and AKT pathways [reviewed in Emmerson et al. (2018)]. The GDF15 protein was recently proposed as a potential marker for radiation response and radiosensitivity in hTERT-immortalized human foreskin fibroblasts (Sandor et al., 2015). Furthermore, it has been reported to contribute to radiation-induced senescence in human aortic endothelial cells (Park et al., 2016) and has been associated with liver fibrosis (Koo et al., 2018).

### Inflammatory Pathways

*CH25H* coding for cholesterol 25-hydroxylase was consistently one of the top3 upregulated genes at different time points and in different strains. The enzyme is important for the synthesis of oxysterol which together with cholesterol promote inflammatory reaction (Gold et al., 2014; Pandak and Kakiyama, 2019) and may contribute to intestinal fibrosis (Raselli et al., 2019). Consistent with this, *IL-6* showed 5-fold upregulation on day 2, increasing to 14-fold at later time points although not sufficient to include it in top25 on day 5 for GS4 or on day 3 for all three cell strains. Pathway analysis showed upregulation of interferon alpha/beta, interferon gamma, and IL-20 family, signaling pathways corroborating that inflammatory signals were induced in the irradiated cultures.

In addition, platelet and neutrophil degranulation pathways and six pathways involving the complement system were upregulated. Notably, the *PTX3* gene coding for the long pentraxin 3 showed consistently strong upregulation in microarrays and qPCR. Pentraxins are important for complement activation (Bonavita et al., 2015; Ma and Garred, 2018). The complement system is an important component of the inflammatory reaction leading to recruitment of blood platelets and neutrophils in the early phases of wound healing (Sinno and Prakash, 2013; Rafail et al., 2015), thus providing a link between radiation-induced inflammation and wound healing (Cazander et al., 2012; Ellis et al., 2018) that may eventually result in fibrotic reaction, analogous to scarring in normal wound healing (Wynn and Ramalingam, 2012).

The *AKCR3* gene coding for atypical chemokine receptor 3 (formerly known as C-X-C chemoreceptor type 7, CXCR7) was among the early upregulated genes in GS4 and five—eightfold upregulated on day 3 in the three strains [mean log2(FC) = 2.8; rank 32]. ACKR3 is a scavenger for CXCL12 and modulates inflammatory signaling through the CXCL12/CXCR4 axis to effector kinases and other targets [reviewed in Giordano et al. (2019); Koenen et al. (2019)]. Interestingly, CXCL12 was part of the 13-gene discovery expression signature associated with the individual risk of subcutaneous fibrosis (Alsner et al., 2007).

### Comparison of Pathways Activated in Different Irradiation Protocols

The MA protocol for studying differentiation showed a high proportion (∼80%) of pathways down-regulated at all three time points, with half of these pathways related to proliferation, cell cycle, cell division, and cell stress and DNA repair. Downregulation of cell stress and DNA repair pathways may appear counter-intuitive but can be explained if the early radiation response is completed by the time RNA was isolated. In contrast with down-regulated pathways, only ∼43% of the upregulated pathways were common to all three time points. These pathways were related to ECM proteins, glycosylation, and interactions of cells with neighboring cells and the ECM, corroborating the association with the radiation-induced phenotypic changes. The differential gene expression after irradiation with two fractions showed that the second fraction enhanced downregulation of most strongly downregulated genes while it had no effect on upregulated genes suggesting that incubation time after the first fraction was of major importance for the transcriptional response.

The expression profile for the fractionated irradiation protocol (AR) for confluent cultures previously used to identify the13-gene predictor of fibrosis risk (later reduced and confirmed for nine genes) (Alsner et al., 2007; Andreassen et al., 2013) was compared with the present protocol. The correlation of differentially expressed genes in the AR protocol applying three fractions to confluent cells was greater with day 5 in the single-dose MA protocol, and the strongest correlation was observed for downregulated genes including those related to the cell cycle. This supports the importance of the first fraction although subsequent fractions may enhance downregulation, whereas the 2 h-interval after the last fraction may not be critical for the radiation-induced expression profile. Upregulated pathways showed some overlap (37/101 significant pathways) between the two protocols, including pathways related to ECM, GAG, inflammation, and cholesterol and bile acid/salts. However, the pathways overrepresented only in the MA protocol (37/74) were dominated by GAG, cell–cell/cell–matrix interactions, and inflammation, whereas the pathways overrepresented only in the AR protocol (27/64) were associated with cholesterol and bile acid/salts, translation, metabolism, and different inflammatory pathways. Thus, although some pathways were common, the two protocols showed considerable differences in the functions of their activated pathways that may represent different aspects of the fibrogenic process.

### Limitations and Conclusion

The main limitation of the present study is the small number of repeat experiments and fibroblast strains. However, the fold change in the expression of the filtered, strongly regulated genes (>4-fold) showed little variation between replicate experiments. A third, independent experiment confirmed the high reproducibility for the same fibroblast strain further supporting that the rate of false discoveries was very low. Differential gene expression in three different strains with additional qPCR experiments showed similar results, corroborating the robustness of the main findings. Pathway analysis grouped tens to hundreds of genes into individual pathways thus reducing the influence of stochastic variation of the expression of individual genes. Only genes and pathways that were upregulated on day three in all three fibroblast strains were considered, thus neglecting variation between individual strains. Pathway analysis is inherently biased toward the available curated pathways and in some cases may contain partially overlapping genes sets. However, the number of Reactome pathways was relatively large (>10^3^) and a wide variety of different pathways categories were represented in the results so that the general expression profiles may be considered to reflect true changes with a high degree of certainty.

The results of this study demonstrate that gene expression profiles after irradiation of exponentially growing skin fibroblasts *in vitro* can be related to radiation-induced differentiation and inflammatory reactions. Furthermore, it suggests signaling mechanisms for phenotypic changes and inflammatory pathways that may be tested in future *in vitro* and *in vivo* studies. The irradiation protocol influences the expression profiles and upregulated pathways which seem to reflect different aspects of the fibrogenic process. Thus the present findings provide a model system and a framework for further hypothesis-based studies of radiation-induced fibrogenesis.

## Data Availability Statement

The raw and normalized data are deposited in the Gene Expression Omnibus (GEO) database (http://www.ncbi.nlm.nih.gov/geo/; accession number GSE147733).

## Author Contributions

CH: experimental design, data analysis, and manuscript writing. CS: microarray and pathway analysis. AS: qPCR, pathway analysis and manuscript writing and editing. FG: manuscript writing and editing and funding. FW: infrastructure and funding. All authors contributed to the article and approved the submitted version.

## Conflict of Interest

The authors declare that the research was conducted in the absence of any commercial or financial relationships that could be construed as a potential conflict of interest.

## Footnotes

1 http://www.ncbi.nlm.nih.gov/geo/
