# Supplementary material for: Gene Expression Profiles Reveal Extracellular Matrix and Inflammatory Signaling in Radiation-Induced Premature Differentiation of Human Fibroblast in vitro
Source: Front Cell Dev Biol. 2021 Feb 18;9:539893. doi: 10.3389/fcell.2021.539893 (PMC7930333; doi:10.3389/fcell.2021.539893)
Supplement: Supplementary file 5 [file Data_Sheet_1.pdf]

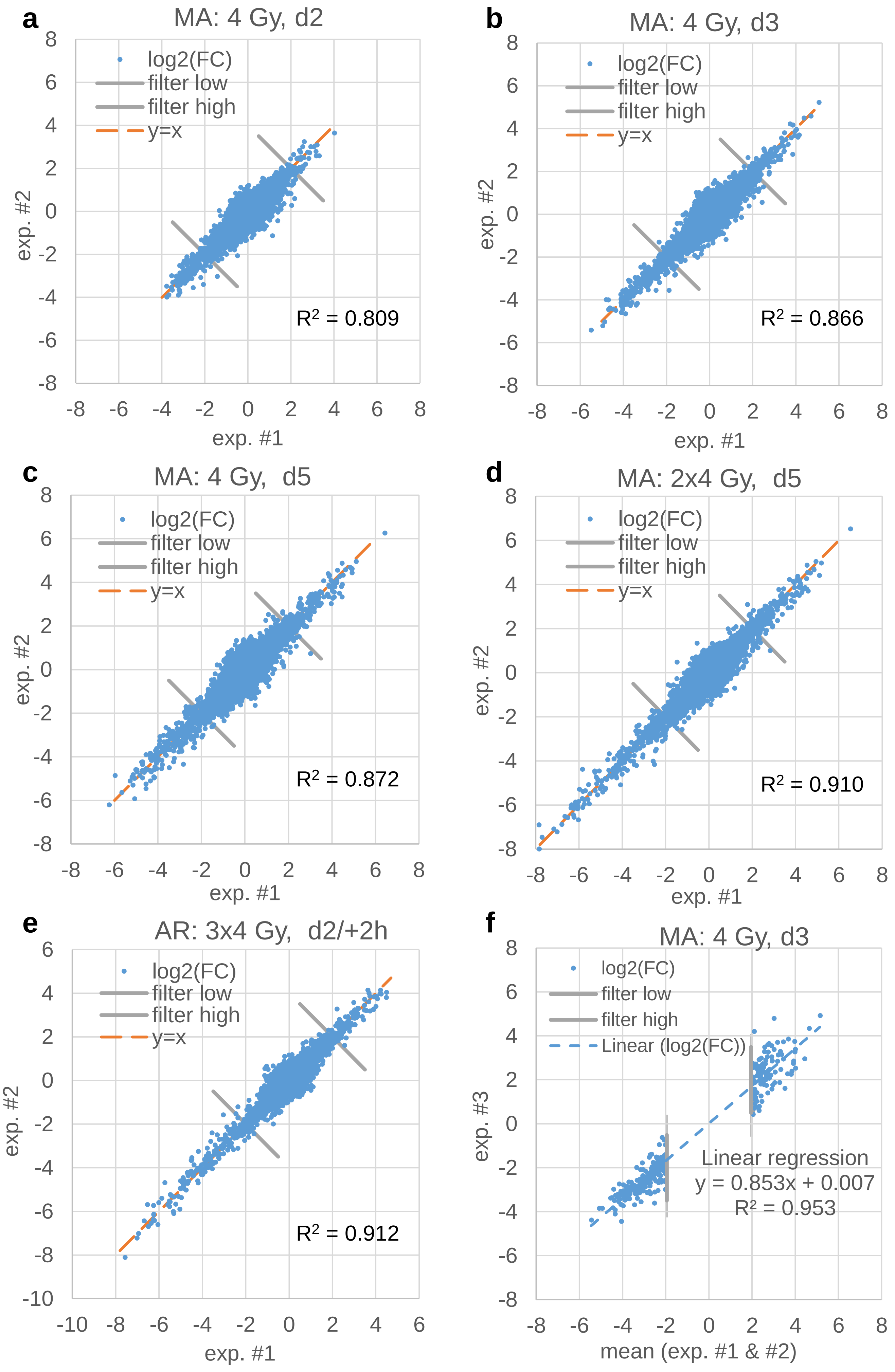

Supplementary Figure 12a-f

Supplementary Figure 1 (previous page). Correlation of radiation-induced log2 fold changes (log2(FC)) in replicate experiments #1 and #2 for values on day 2 (a), day 3 (b), day 5 (c) after irradiation of exponentially growing fibroblasts with 1×4 Gy on day 0 (Mannheim protocol, MA), day 5 after the first fraction of 2×4 Gy given on day 0 and day 3 (d), and irradiation with 3×4 Gy given on day 0, 1, and 2, with RNA isolated 2h after the last fraction (Aarhus protocol, AR) (e). The R^2 values in a-e are for orthologonal regression of the filtered genes assuming the same variance in the two experiments. The correlation of log2(FC) values in experiment #3 with the mean log2(FC) values from #1 and #2, including only filtered genes with mean log2(FC) >2 or < -2 (f). The R^2 value in the latter diagram is for linear regression, assuming a smaller variance for the mean values of exp. #1 and #2 than for exp. #3.

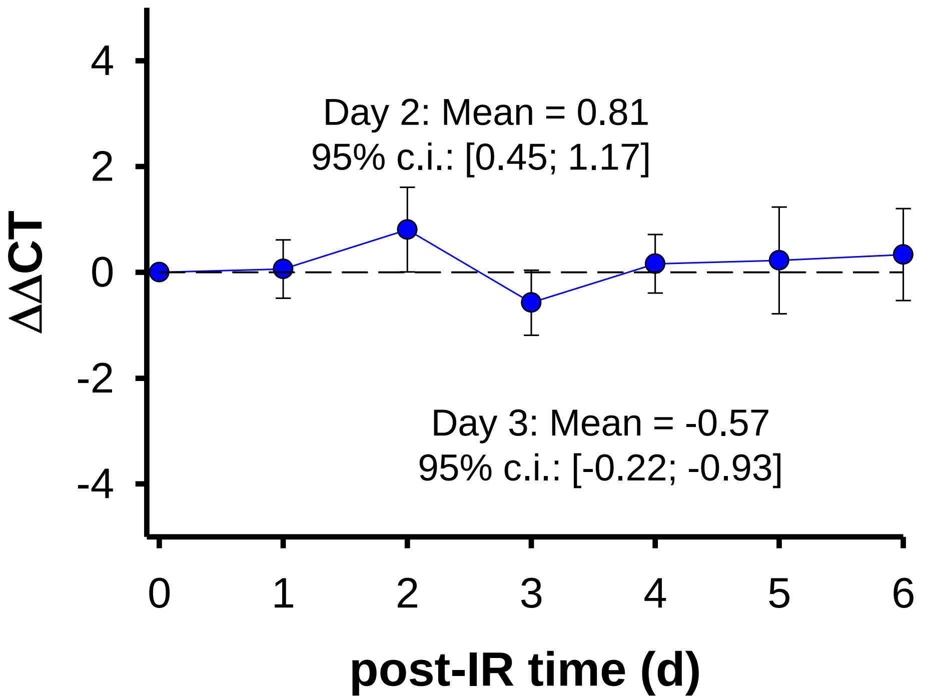

Supplementary Figure S2. Expression levels of the control gene, *ACTB*, as function of time after irradiation. The symbols represent mean ΔΔCt values with standard errors from five qPCR plates for each of the three fibroblast strains, GS3, GS4, and GS5. ANOVA yielded significant differences for day 2 and day 3 (mean values and 95% confidence intervals are shown). The variations would result in a small underestimation expression levels for the genes of interest on day 2 and a small overestimation on day 3.

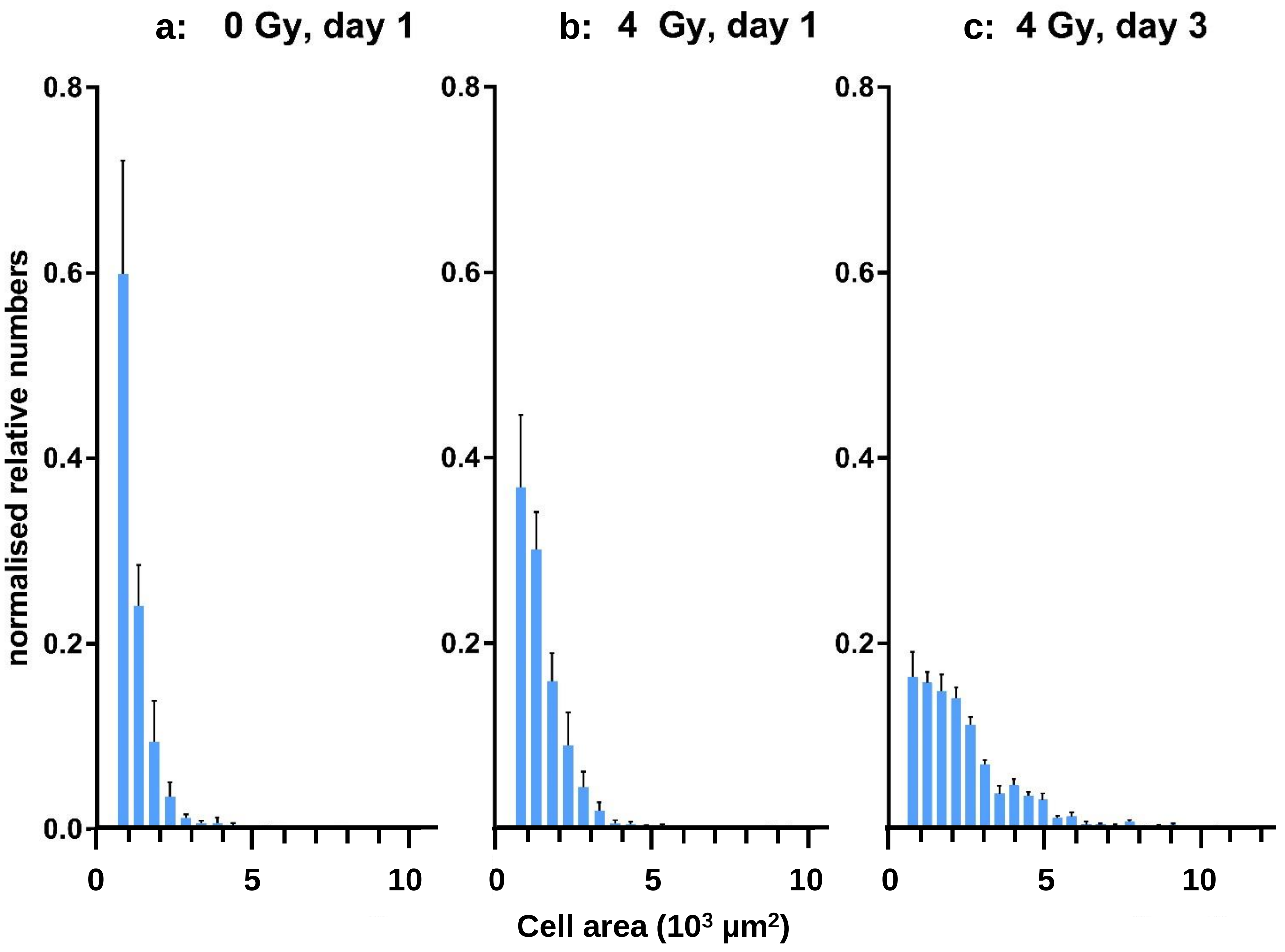

Supplementary Figure S3. Normalised distributions of fibroblast cell sizes in GS4 fibroblast cultures on day 1 without irradiation (a) and on day 1 (b) and day 3 (c) after irradiation with a single dose of 4 Gy. The histograms show mean values and standard errors of n=3 repeat experiments. For each experiment and condition, four microscopic images were taken with a 10× objective and a total of 300-600 cells per condition were analysed with Image J software. The median areas increased significantly from day 1 to day 3: the mean values (± s.e.m.) of the individual medians in each experiment increased from 934±131 µm^2 (0 Gy, day 1) to 2161±46 µm^2 (4 Gy, day 3); p=0.02 (n=3, paired t-test).

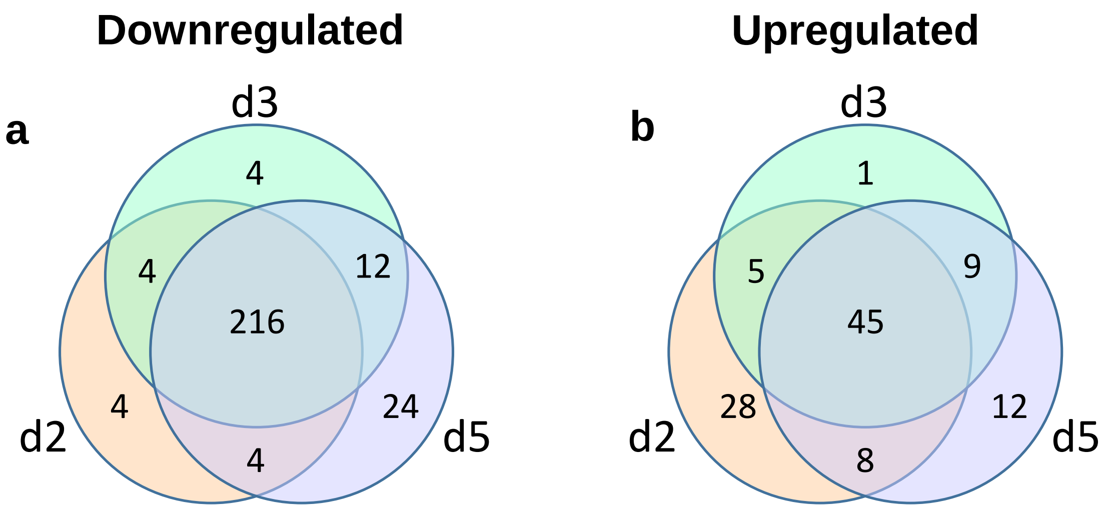

Supplementary Figure S4. Venn diagram for the number of filtered genes down- and up-regulated on day 2, 3, and 5, in experiments # 1 and #2.

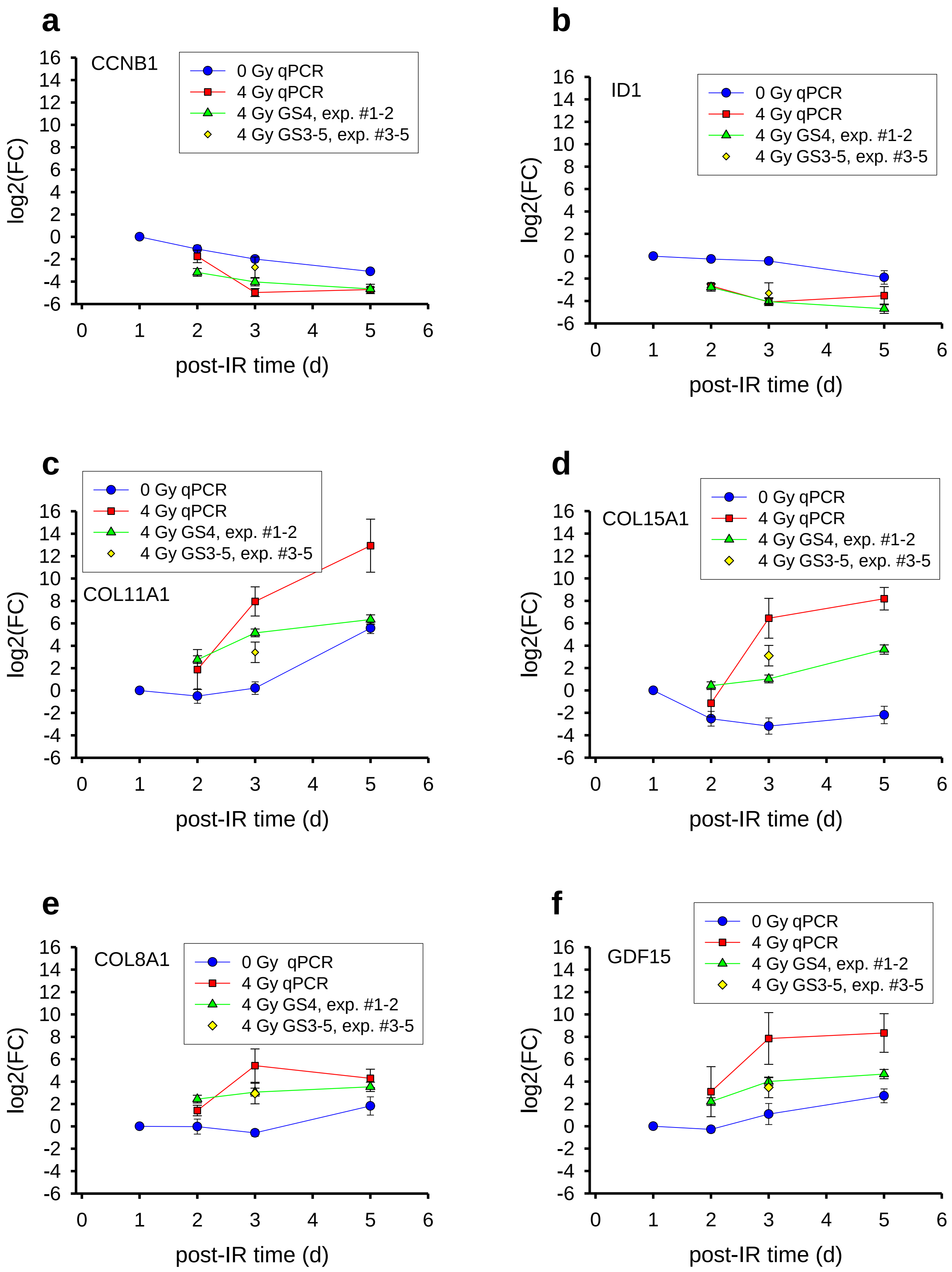

Supplementary Figure S5a-f

g

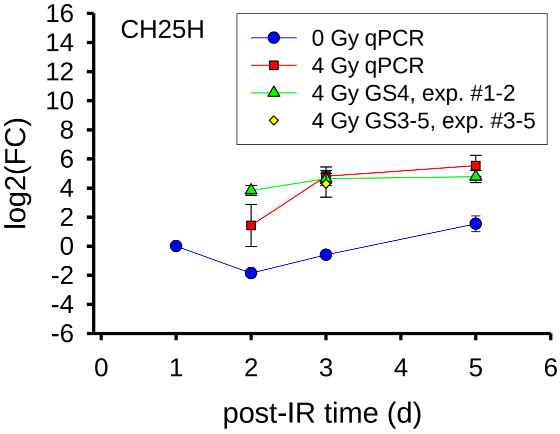

h

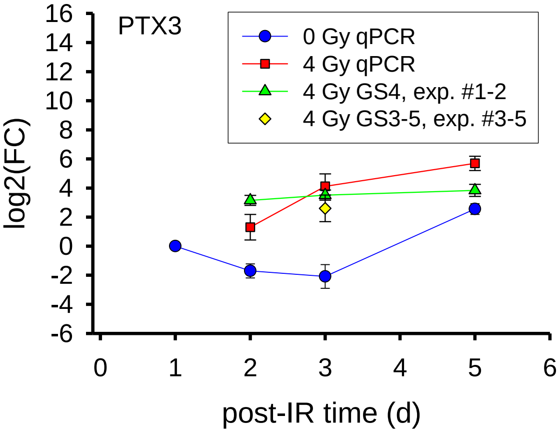

i

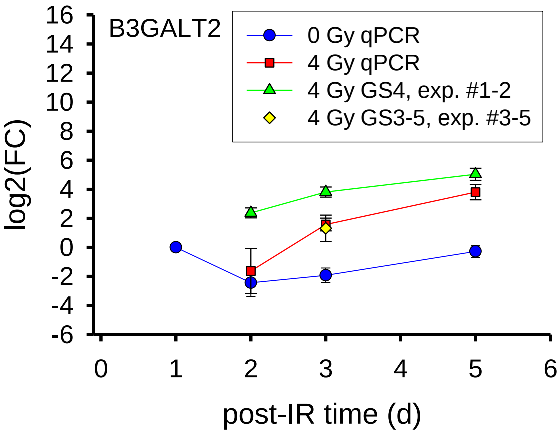

j

Gene	P (d2)	P (d3)	P (d5)
CCNB1	0.40	0.004*	0.047*
ID1	0.099	0.062	0.080
COL8A1	0.22	0.068	0.014*
COL11A1	0.24	0.013*	0.082
COL15A1	0.25	0.061	0.023*
BGALT2	0.54	0.003*	0.003*
GDF15	0.25	0.041*	0.037*
CH25H	0.15	0.005*	0.002*
PTX3	0.12	0.011*	0.004*

k

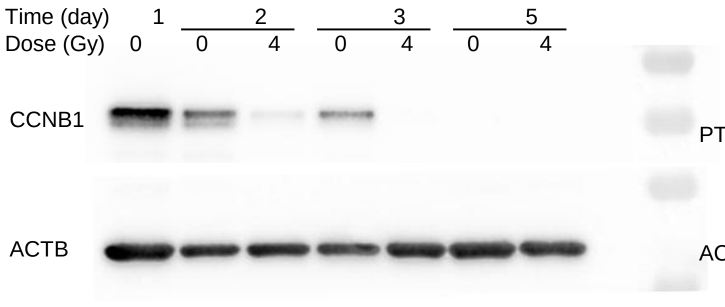

k

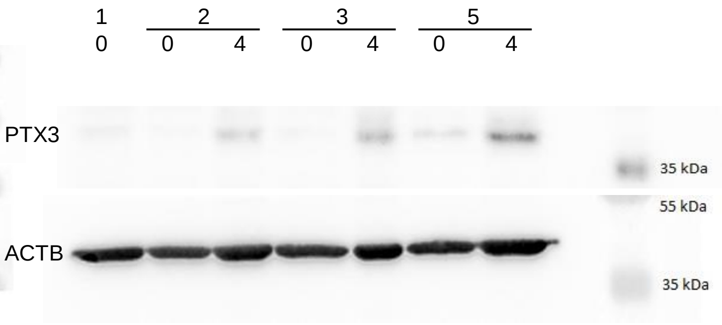

m

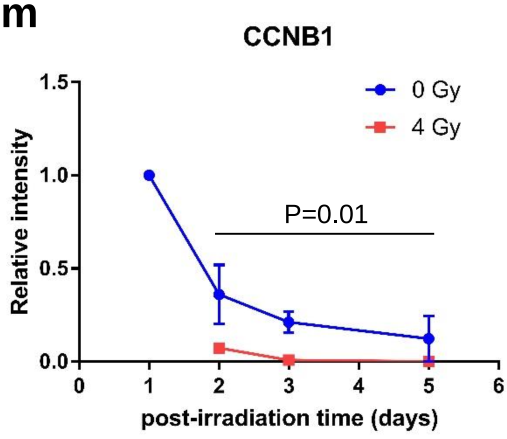

n

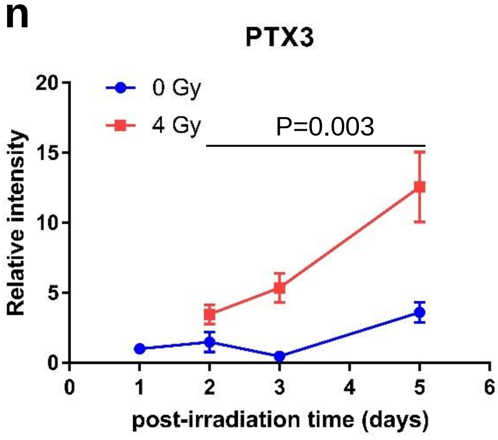

Supplementary Figure S5g-n

Supplementary Figure S5 (previous two pages). Additional validation experiments including unirradiated controls for day 1, 2, 3, and 5. Mean values and standard errors from n=3 independent experiments with GS3, GS4, and GS5, are shown (some error bars are smaller than the symbols).qPCR results for none genes are shown in (a-i). Microarray data from experiment #1-2 and #3-5 are included for comparison. P-values for the difference between irradiated and unirradiated samples are shown in the table (paired t-test, N=6), significant values are indicated by an asterisk. Western blots for CCNB1 and PTX3 are shown in (j-k) and quantified in (l-m). P-values for paired t-tests were performed for irradiated versus unirradiated samples on day 2, 3, and 5, together.

a

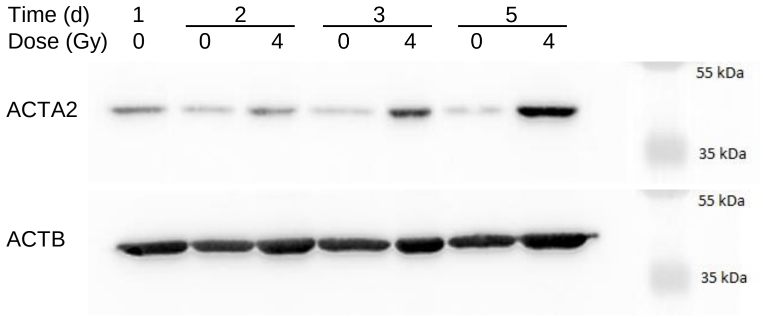

b

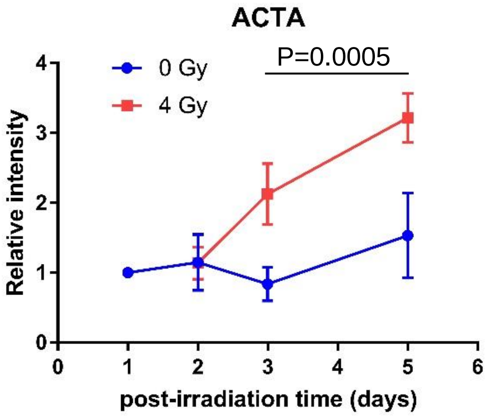

Supplementary Figure S6a,b. Western blots for ACTA2 were obtained in three independent experiments with GS3, GS4, and GS5, respectively. GS3 is shown (a). All three Western blots were quantified and mean values and standard errors (n=3) are shown in (b). P-values for paired t-tests were performed for irradiated versus unirradiated samples on day 3, and 5, together.

ACTA2: 3 frames per dose and time point

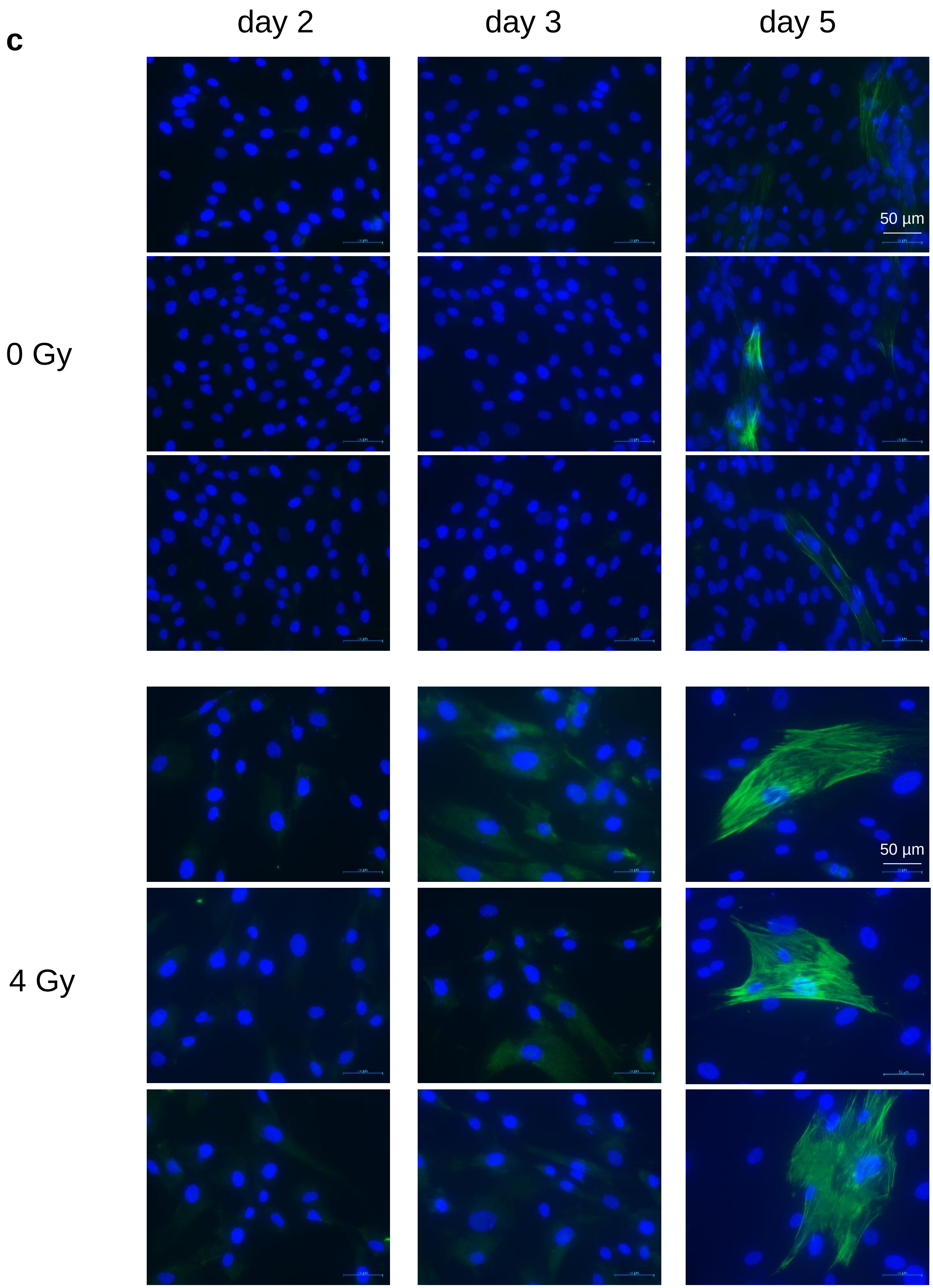

Supplementary Figure S6c

Supplementary Figure S6c (previous page). Fluorescence microscopy for ACTA2 expression in GS4 on day 2, 3, and 5, are shown for unirradiated and irradiated samples (c). Three image frames from a single microscope slide are shown for each day. Green fluorescence (FITC) shows α-smooth muscle actin with cell nuclei in blue stain (DAPI). Weak, diffuse cytoplasmic staining is seen in the cytoplasm at early time points but the intensity increases on day 3 after 4 Gy, and on day 5 stress fibres appear in small clusters of adjacent cells, especially in irradiated cultures but a minor extent is also observed in unirradiated cultures. The results are from a single experiment but confirm similar observations of ACTA2 stress fibres in cell clusters from previous experiments.

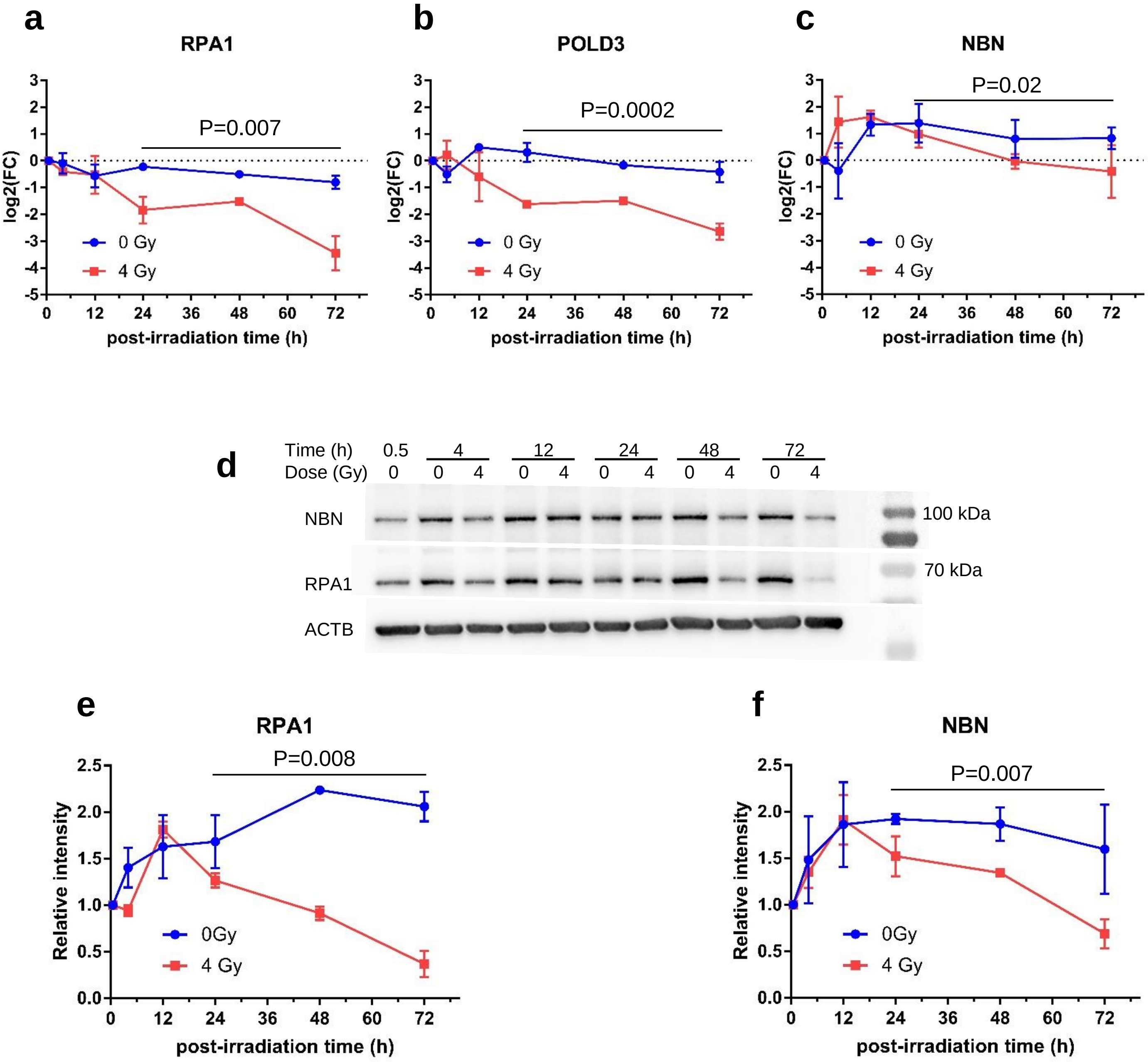

Supplementary Figure S7. Additional validation experiments including unirradiated controls for time points 0.5h, 4h, 12h, 24h (day 1), 48h (day 2), and 72h (day 3) for cell stress- and repair-related genes *RPA1*, *POLD3*, and *NBN*. Mean values and standard errors from n=3 independent experiments with GS3, GS4, and GS5, are shown (some error bars are smaller than the symbols). qPCR results for none genes are shown in (a-c). P-values for the difference between irradiated and unirradiated samples are shown in the table (paired t-test, N=6). Western blots for RPA1 and NBN are shown in (d) and quantified in (e-f). P-values for paired t-tests were performed for irradiated versus unirradiated samples on day 1, 2, and 3, together.
